# Lipids and the Permeability and Antimicrobial Barriers of the Skin

**DOI:** 10.1155/2018/5954034

**Published:** 2018-09-02

**Authors:** Philip W. Wertz

**Affiliations:** Professor Emeritus, University of Iowa, Iowa City, IA 52240, USA

## Abstract

The primary purpose of the epidermis of terrestrial vertebrates is to produce the stratum corneum, which serves as the interface between the organism and the environment. As such, the stratum corneum provides a permeability barrier which both limits water loss through the skin and provides a relatively tough permeability barrier. This provides for a degree of resistance to mechanical trauma and prevents or limits penetration of potentially harmful substances from the environment. The stratum corneum consists of an array of keratinized cells embedded in a lipid matrix. It is this intercellular lipid that determines the permeability of the stratum corneum. The main lipids here are ceramides, cholesterol, and fatty acids. In addition, the skin surface of mammals, including humans, is coated by a lipid film produced by sebaceous glands in the dermis and secreted through the follicles. Human sebum consists mainly of squalene, wax monoesters, and triglycerides with small proportions of cholesterol and cholesterol esters. As sebum passes through the follicles, some of the triglycerides are hydrolyzed by bacteria to liberate free fatty acids. Likewise, near the skin surface, where water becomes available, some of the ceramides are acted upon by an epithelial ceramidase to liberate sphingosine, dihydrosphingosine, and 6-hydroxysphingosine. Some of the free fatty acids, specifically lauric acid and sapienic acid, have been shown to have antibacterial, antifungal, and antiviral activity. Also, the long-chain bases have broad spectrum antibacterial activity.

## 1. Epidermal Lipid Composition Alters with Differentiation

As cells in the epidermis move from the basal layer toward the skin surface, they become larger and flatter. They progressively synthesize keratins and other proteins, and they synthesize and accumulate lipids [1]. The composition of the lipids alters with differentiation until the final unique lipid mixture in the stratum corneum is obtained. This consists mainly of ceramides (50% by weight), cholesterol (27%), and fatty acids (10%) with small amounts of cholesterol oleate and cholesterol sulfate [1]. It is this lipid mixture that determines the quality of the permeability barrier of the skin. This barrier limits water loss through the skin, and this has permitted the evolution of life as we know it on dry land [2]. This permeability barrier also protects the underlying tissue against potentially toxic substances from the environment. Some of the stratum corneum lipids also serve as substrates for the enzymatic production of potent antimicrobials at the skin surface. These antimicrobials are the long-chain bases -- sphingosine, dihydrosphingosine, and possibly 6-hydroxysphingosine. The structure of cholesterol has long been known, and the free fatty acids in stratum corneum are straight-chained saturated species ranging from 14- to 28-carbons in length with the C-22, C-24, and C-26 entities being the most abundant [3]. However, it has taken decades to fully define the changes in lipid composition accompanying keratinization and to identify and determine the structures of the ceramides of the stratum corneum.

In 1932, Kooyman demonstrated that human epidermal basal cells contained phospholipids as the main lipid constituents, while stratum corneum contained mainly neutral lipids including cholesterol [4]. At this time ceramides had not yet been identified in epidermis.

Another early demonstration that epidermal lipid composition alters with differentiation came from the work of VJW Long [5, 6]. Long cut six horizontal slices through cow snout epidermis. Each slice was 0.2 mm thick. Lipids were then extracted and analyzed. The lowest slice contained some dermis, the basal, and spinous cells, and the outermost slice contained the stratum corneum and some granular cells. Phospholipids, cholesterol, and free fatty acids were analyzed. Phospholipids increased from the basal layer through the next two slices and then decreased sharply as the stratum corneum was approached. Cholesterol and fatty acids were very low in the innermost four slices and showed a slight increase in the slice below the stratum corneum and a larger increase in the outermost slice containing the stratum corneum. In 1965, Nicolaides had identified ceramides as a major neutral lipid at the cell surface [7]. This identification was based on infrared spectral data and was included in a footnote added in proof (personal communication). Long was apparently not aware of this and did not comment on ceramides.

**Figure 1:**
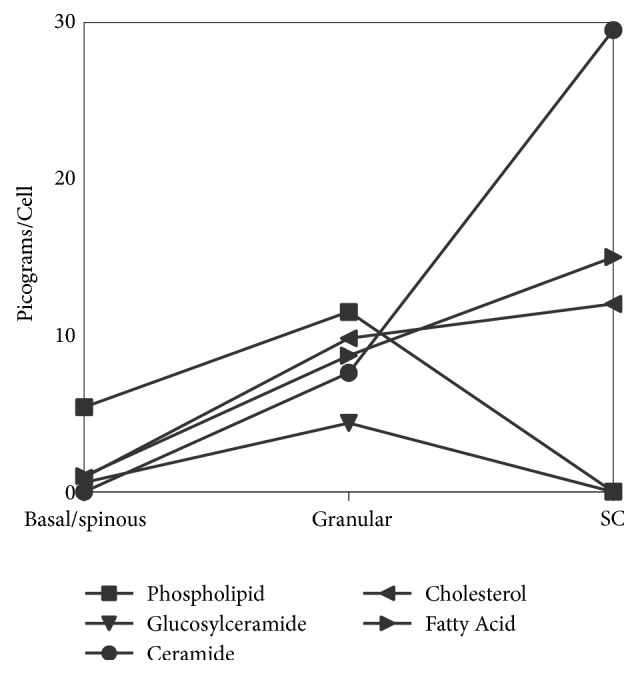
Alteration of epidermal lipid content and composition with differentiation based on data from Gray and Yardley [8].

In 1975, Gray and Yardley [8] isolated different populations of keratinocytes from porcine epidermis by progressive trypsinization. One fraction consisted of basal and spinous cells, one of granular cells, and the third fraction was stratum corneum. They found that the basal/spinous cell fraction contained mostly phospholipids with small amounts of cholesterol and glucosylceramides. The granular layer fraction contained higher proportions of cholesterol and glucosylceramides and lower proportions of phospholipids. The stratum corneum contained the highest proportions of cholesterol and ceramides. In the stratum corneum, phospholipids represented 0.1% of the lipid mass compared to 62.3% in the basal/spinous cell fraction. These findings are summarized in Figure 1.

## 2. Ceramide Structures

Gray and his collaborators also demonstrated that the ceramides, found mainly in the stratum corneum, and the glucosylceramides, found in the viable epidermis, are structurally heterogenous [9]. They identified building blocks of the sphingolipids to include straight-chained saturated fatty acids ranging from 14- to 30-carbons in length, *α*-hydroxyacids, sphingosines, dihydrosphingosines, and phytosphingosines. Porcine epidermis was very similar to human epidermis in terms of the lipid compositions.

In 1983, the first complete set of epidermal ceramide structures was published [10]. The porcine epidermal ceramides consisted of six chromatographically distinct fractions, and each had a defined structure. These structural entities were numbered 1 through 6 in order of increasing polarity. Ceramide 1 was most unusual. It consisted of 30- to 34-carbon long *ω*-hydroxyacids amide-linked to a mixture of sphingosines and dihydrosphingosines with linoleic acid ester-linked to the *ω*-hydroxyl group. Ceramide 2 consisted of straight-chained, saturated fatty acids amide-linked to sphingosines and dihydrosphingosines. The fatty acids in ceramide 2 were mostly 24-, 26-, and 28-carbons in length. Ceramide 3 contained the same long, saturated fatty acids amide-linked to a mixture of phytosphingosines. Ceramides 4 and 5 both contained *α*-hydroxyacids amide-linked to sphingosines and dihydrosphingosines. They differed in that ceramide 4 contained 24- through 28-carbon *α*-hydroxyacids while ceramide 5 contained *α*-hydroxypalmitic acid. The difference in hydroxyacid chain lengths results in chromatographic separation of these two ceramide fractions even though they both contain *α*-hydroxyacids amide-linked to sphingosine and dihydrosphingosine. Ceramide 6 contained the longer variety of *α*-hydroxyacids amide-linked to phytosphingosines. Determination of these structures has been of significance for the cosmetics industry, transdermal drug delivery, and dermatology.

While the numbering system of nomenclature worked adequately for porcine ceramides, this did not carry over to human epidermal ceramides, where the situation was more complicated. Motta et al. [11] proposed a new nomenclature system to address this problem. In the Motta system, the amide-linked fatty acid is designated as N, A, or O, for normal fatty acid, *α*-hydroxyacid, or *ω*-hydroxyacid. The long-chain base was indicated as S or P for sphingosine or phytosphingosine. Sphingosine and dihydrosphingosine always occurred together, so no separate designation was proposed for dihydrosphingosine. A prefix E indicated an ester-linked fatty acid on the hydroxyl group of an *ω*-hydroxyacid. In normal healthy epidermis, this has always been predominantly linoleate. With the subsequent identification of 6-hydroxysphingosine in human epidermal ceramides H has been added to this system to indicate this base [12]. Some investigators have made the distinction between sphingosine and dihydrosphingosine by using S for the former only and DS for the latter [13–15]. In the original Motta proposal, the stratum corneum ceramides with a base designated as S actually contained a mixture of sphingosine and dihydrosphingosine. These species did not separate by conventional TLC. To describe such mixed-base ceramide fractions, it is proposed to use S/DS. In this system, the porcine ceramides become CER EOS/DS (7.1% of total ceramide by weight), CER NS/DS (40.7%), CER NP (15.6%), CER A_l_S/DS (11.6%), CER A_s_S/DS (10.1%), and CER AP (14.9%). The subscripts l and s have been added to indicate long *α*-hydroxyacids and short *α*-hydroxyacids.

**Figure 2:**
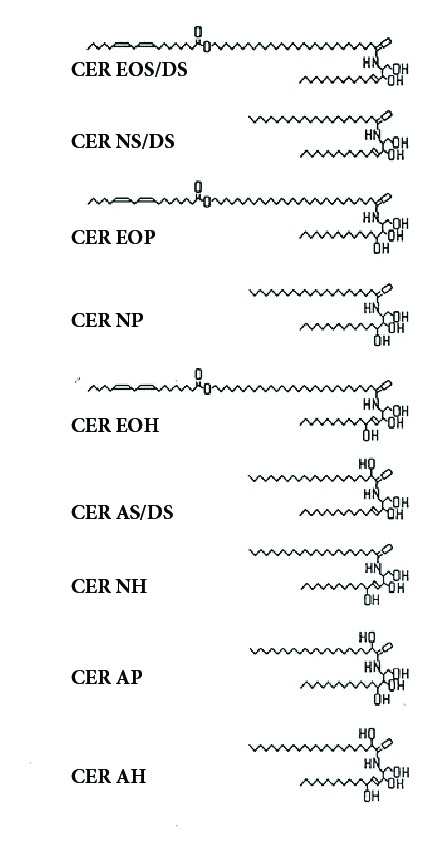
Structures of free ceramides from human stratum corneum [16].

The first complete set of human ceramide structures was published in 2003 [16]. These consisted of CER EOS/DS (8.3%), CER EOP (6.4%), CER EOH (5.0%), CER NS/DS (20.5%), CER NP (18%), CER NH (16.0%), CER AS/DS (3.7%), CER AP (8.6%), and CER AH (12.9%). Again, sphingosine was always accompanied by dihydrosphingosine as indicated by S/DS. These structures are shown in Figure 2.

## 3. Mass Spectrometry and Individual Ceramide Structures

The introduction of high performance liquid chromatography in combination with electrospray ionization and tandem mass spectroscopic methodology for analysis of stratum corneum ceramides has led to the identification and quantitation of individual molecular species [19]. In this initial study, 182 molecular entities were identified. Similar methodology has been used to examine seasonal variation in ceramides linked to changes in barrier function in acne patients [20]. This methodology has also been used to examine stratum corneum ceramides in healthy Chinese subjects [15].

Liquid chromatography combined with dynamic multipole reaction monitoring of mass spectrometry has been used to study the effects of aging on stratum corneum ceramides. A total of 93 biomarkers were identified to indicate differences between children and adults [14].

Mass spectrometry has also been used to study stratum corneum lipids in Netherton syndrome [21]. These patients have severe permeability barrier disfunction. In general, it was found that free fatty acids were shorter than normal and contained more monounsaturated species. The ceramides contained reduced proportions of long-chain fatty acids and elevated proportions of shorter fatty acids.

The product of the PNPLA1 gene is a transacylase necessary for the formation of *ω*-O-acylceramides [22]. Mass spectral analysis of ceramides from PNPL1 mutants revealed the defect in acylceramide synthesis [23]. PNPLA1 knockout mice die shortly after birth due to a severely defective permeability barrier.

## 4. Covalently Bound Ceramides

Covalently bound *ω*-hydroxyceramides were first reported in porcine stratum corneum [24] and were subsequently found in human [17] and murine stratum corneum [18, 25].

**Figure 3:**
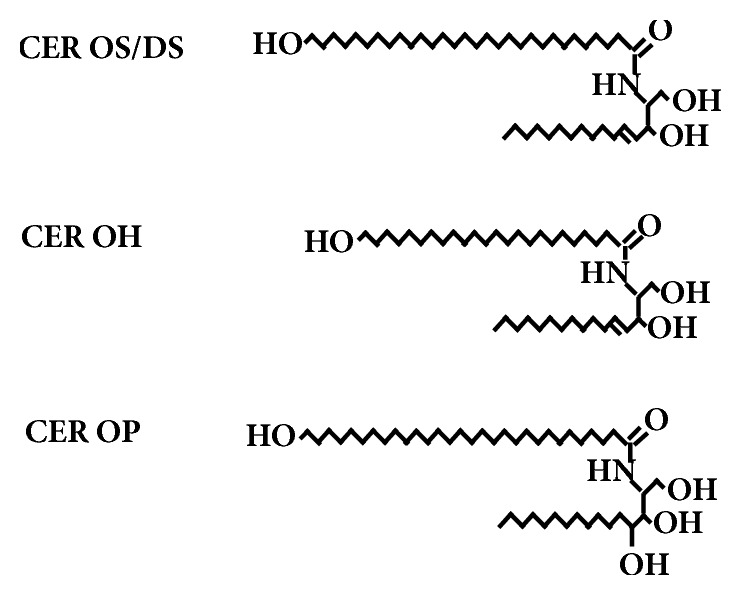
Structures of the covalently bound *ω*-hydroxyceramides from porcine (OS/DS) and human (OS/DS, OH and OP) stratum corneum [12, 17, 18].

Ceramides are synthesized in the viable portion of the epidermis and are immediately glucosylated to form glucosylceramides. These glycolipids are associated with lamellar granules [26]. In the uppermost granular cells, the bounding membrane of the lamellar granule fuses into the cellular plasma membrane and the contents of the lamellar granule are extruded into the intercellular space [27]. In addition to glucosylceramides and sphingomyelin, the lamellar granules deliver to the intercellular space acid sphingomyelinase and glucocerebrosidase [28–30]. These enzymes convert the sphingomyelin and glucosylceramides into ceramides that pass into the stratum corneum. The most abundant of the glucosylceramides are the *ω*-hydroxyacid-containing glucosylceramides with linoleate ester-linked to the *ω*-hydroxyl group [31, 32]. Based on the amounts of acylglucosylceramide associated with the lamellar granules and the amounts of corresponding acylceramides that can be extracted from the stratum corneum, it has been suggested that about two-thirds of the lamellar granule-associated acylglucosylceramide is in the bounding membrane, and the remaining one-third is in the internal lamellae of the lamellar granule [33]. The bounding membrane of the lamellar granule fuses into the cell plasma membrane as the proteins of the cornified envelope are being deposited [27]. The acylglucosylceramide at the cell periphery becomes deglycosylated, and the linoleate is removed. The resulting *ω*-hydroxyceramides become ester-linked to acidic amino acid side groups at the surface of the cornified envelope [17, 24]. This process involves lipoxygenase action on the linoleate tail [34], action of epoxide hydrolases [35], glucocerebrosidase [30], and possibly transglutaminase [36]. The result is covalently bound CER OS/DS [17, 24], CER OP [37], and CER OH [12, 17]. The structures of the covalently bound *ω*-hydroxyceramides are presented in Figure 3.

It has been suggested that the covalently bound lipid layer provides a template upon which the free lipids form intercellular lamellae [38]. There is also evidence that the covalently bound lipids may play a role in cell-cell cohesion [39]. This layer of covalently bound lipid appears in transmission electron micrographs as a continuous 4 nm thick membrane and persists after extensive extraction of stratum with chloroform:methanol mixtures to remove free lipids [40]. Gray and Yardley noted in 1975 that the cell plasma membrane appeared to persist from the basal keratinocyte through the viable epidermis and into the stratum corneum as judged by transmission electron microscopy [8]. However, they pointed out that this structure in the stratum corneum must be different from the plasma membrane in the viable epidermis because there were no phospholipids in the stratum corneum. Also, a freeze-fracture study employing filipin as a probe to detect cholesterol in membranes noted that the intercellular lamellae of stratum corneum contained abundant cholesterol, but the lamella adjacent to the cornified envelope, now known to be a layer of covalently bound *ω*-hydroxyceramides, contained none [41].

## 5. Metabolism of Ceramides

Palmitic acid is synthesized from acetyl-coenzyme A on the cytosolic acyl carrier protein complex [42]. Palmitic acid can then be released as the free fatty acid or transferred to coenzyme A. Chain extension starting with palmitoyl-coenzyme A can take place in the endoplasmic reticulum [43]. If a chain is extended long enough to span the endoplasmic reticulum, it can be hydroxylated on the terminal carbon to produce an *ω*-hydroxyacid-coenzyme A [44]. Palmitoyl-coenzyme A can also be hydroxylated on the 2 position to produce *α*-hydroxyacid-coenzyme A [45].

Serine-palmitoyltransferase is the rate-limiting enzyme in sphingosine, and thereby sphingolipid, biosynthesis [46]. It condenses serine with palmitoyl-coenzyme A to produce 3-keto-dihydrosphingosine. The ketone group in this intermediate is reduced in an NADPH dependent reaction to produce dihydrosphingosine which is rapidly condensed with a fatty acid-coenzyme A by a ceramide synthase to produce a ceramide. A trans double bond can then be introduced between carbons 4 and 5 of the base to produce a sphingosine-containing ceramide [45]. Hydroxylations of the long-chain base component can produce phytosphingosine or 6-hydroxysphingosine [45].

As noted elsewhere, ceramidases can hydrolyze ceramides to liberate free long-chain bases.

## 6. Sebaceous Lipids

Sebum is the product of holocrine glands in the dermis and is secreted through follicles onto the skin surface [47, 48]. Sebum composition is species specific. Human sebum consists of squalene, wax monoesters, triglycerides, and small proportions of cholesterol and cholesterol esters. As sebum flows outward through the follicles, the triglycerides undergo partial hydrolysis by bacterial lipases to produce free fatty acids. The fatty acids reaching the skin surface include saturated species ranging from 7- to 22-carbons in length with palmitic acid being the most abundant [49]. It is notable that this includes lauric acid (C12:0). Monounsaturates are found in the range of 14- through 18-carbons with sapienic acid, C16:1Δ6, being the most abundant. There are 18-carbon dienoic species that represent less than 1% of the total fatty acids. One of these is linoleic acid derived from the diet. The other is the Δ 5,8 isomer [50].

## 7. Antimicrobial Fatty Acids and Long-Chain Bases at the Skin Surface

In 1942, Burtenshaw demonstrated that lipids collected from the human skin surface could kill* Staphylococcus aureus* [51]. The composition of the skin surface lipids was not completely known at this time, but it was suggested that fatty acids could be the active components. This view became widely accepted. In 1947, Weitkamp et al. fractionated human sebaceous fatty acid by fractional distillation [49]. They tested each fraction for antifungal activity against* Microsporum audouini*, an organism that causes ringworm of the scalp in infants [52]. They found that the short chain saturated species (C7:0, C9:0, C11:0, and C13:0) were active against this fungus. Sebum secretion is very low in children prior to the onset of puberty [52]. This explains why “cradle cap” recurs in infants and children until the onset of puberty, after which the infection disappears and never returns.

Lauric acid, sphingosine, and dihydrosphingosine have been shown to be antibacterial against a number of organisms. Lauric acid was shown to be a uniquely potent antibacterial against a range of gram positive bacteria but not gram negatives [53]. Sphingosine and dihydrosphingosine were shown to be active against* S. aureus*,* Streptococcus pyogenes*,* Micrococcus luteus*,* Propionibacterium acnes*,* Brevibacterium epidermidis,* and* Candida albicans *[54].

Only recently were fatty acids actually isolated from the human skin surface tested for antibacterial activity [55]. It turns out that lauric acid (C12:0) and sapienic acid (C16:1Δ6) were potent antibacterials versus* S. aureus*. Octanoic acid, decanoic acid, myristic acid, and palmitic acid displayed bacteriostatic activity but were not bactericidal under the conditions of the testing. Recent studies have shown that lauric acid is active against gram positive bacteria including* S. aureus*,* Streptococcus mitis*,* Streptococcus sanguinis*,* Corynebacterium bovis*,* Corynebacterium striatum,* and* Corynebacterium jeikeium*. It was not active against gram negative* Escherichia coli*,* Serratia marcescens,* or* Pseudomonas aeruginosa*, but it was active against* Fusobacterium nucleatum [56]*. Sapienic acid was active against S.* sanguinis*,* S. mitis*,* F. nucleatum,* and* Porphyromonas gingivalis*. It was inactive against* E. coli*,* S. marcescens*,* P. aeruginosa*,* C. bovis*,* C. striatum,* and* C. jeikeium* [56].

Free sphingosine, dihydrosphingosine, and 6-hydroxysphingosine have been detected in stratum corneum [57–61]. An epidermal ceramidase has been identified that could liberate free long-chain bases from some of the ceramides. There is some evidence that covalently bound ceramides could be among the ceramidase substrates. In porcine stratum corneum, there is a major amount of covalently bound CER OS/DS and a small amount of covalently bound *ω*-hydroxyacid [17, 24]. The latter probably reflects ceramidase action on covalently bound CER OS/DS with liberation of sphingosine and dihydrosphingosine and retention of the *ω*-hydroxyacid. When young pigs are fed an essential fatty acid deficient diet the transepidermal water loss (TEWL) increases progressively over some weeks [61]. The increase in TEWL is accompanied by a decrease in covalently bound CER OS/DS and a corresponding increase in covalently bound *ω*-hydroxyacid. This may provide a mechanism to ward off infection under conditions where the permeability barrier is compromised.

Sphingosine and dihydrosphingosine have been found to be antibacterial against* E. coli*,* S. mitis*,* S. aureus*,* S. sanguinis*,* C. bovis*,* C. striatum*,* C. jeikeium*, and* F. nucleatum*. It was inactive versus* S. marcescens* and* P. aeruginosa [56]*.

## 8. Summary

In the epidermis, lipid composition alters dramatically as a function of differentiation. Ultimately, a mixture of cholesterol, fatty acids, and ceramides is end products of the differentiation process. These lipids underlie the permeability barrier of the stratum corneum.

Free fatty acids liberated from sebaceous triglycerides serve as selective antimicrobial agents at the skin surface. Free long-chain bases derived from ceramides in the stratum corneum serve as broader acting antimicrobial agents at the surface of the skin and its appendages. Under some conditions, it may be useful to provide exogenous supplies of these lipids to prevent or treat infection.
